# The nasal microbiota is a potential diagnostic biomarker for sepsis in critical care units

**DOI:** 10.1128/spectrum.03441-23

**Published:** 2024-06-12

**Authors:** XiLan Tan, Haiyue Liu, Wen Qiu, Zewen Li, Shuang Ge, Yuemei Luo, Nianyi Zeng, Manjun Chen, Qiqi Zhou, Shumin Cai, Jun Long, Zhongran Cen, Jin Su, Hongwei Zhou, Xiaolong He

**Affiliations:** 1 Division of Infection Medicine, Zhujiang Hospital, Southern Medical University, Guangzhou, China; 2 State Key Laboratory of Organ Failure Research, Division of Laboratory Medicine, Zhujiang Hospital, Southern Medical University, Guangzhou, China; 3 Xiamen Key Laboratory of Genetic Testing, The department of laboratory medicine, The First Affiliated Hospital of Xiamen University, School of Medicine, Xiamen University, Xiamen, China; 4 Microbiome Medicine Center, Department of Laboratory Medicine, Zhujiang Hospital, Southern Medical University, Guangzhou, Guangdong, China; 5 Department of Intensive Care Medicine, Nanfang Hospital, Southern Medical University, Guagnzhou, China; 6 Division of Intensive Care Medicine, Zhujiang Hospital, Southern Medical University, Guangzhou, China; 7 Chronic Airways Diseases Laboratory, Department of Respiratory & Critical Care Medicine, Nanfang Hospital, Southern Medical University, Guangzhou, China; University of Arkansas Fayetteville, Fayetteville, Arkansas, USA

**Keywords:** sepsis, nasal microbiota, gut microbiota, 16S rRNA

## Abstract

This study aimed to characterize the composition of intestinal and nasal microbiota in septic patients and identify potential microbial biomarkers for diagnosis. A total of 157 subjects, including 89 with sepsis, were enrolled from the affiliated hospital. Nasal swabs and fecal specimens were collected from septic and non-septic patients in the intensive care unit (ICU) and Department of Respiratory and Critical Care Medicine. DNA was extracted, and the V4 region of the 16S rRNA gene was amplified and sequenced using Illumina technology. Bioinformatics analysis, statistical processing, and machine learning techniques were employed to differentiate between septic and non-septic patients. The nasal microbiota of septic patients exhibited significantly lower community richness (*P* = 0.002) and distinct compositions (*P* = 0.001) compared to non-septic patients. *Corynebacterium*, *Staphylococcus*, *Acinetobacter*, and *Pseudomonas* were identified as enriched genera in the nasal microbiota of septic patients. The constructed machine learning model achieved an area under the curve (AUC) of 89.08, indicating its efficacy in differentiating septic and non-septic patients. Importantly, model validation demonstrated the effectiveness of the nasal microecological diagnosis prediction model with an AUC of 84.79, while the gut microecological diagnosis prediction model had poor predictive performance (AUC = 49.24). The nasal microbiota of ICU patients effectively distinguishes sepsis from non-septic cases and outperforms the gut microbiota. These findings have implications for the development of diagnostic strategies and advancements in critical care medicine.

**IMPORTANCE:**

The important clinical significance of this study is that it compared the intestinal and nasal microbiota of sepsis with non-sepsis patients and determined that the nasal microbiota is more effective than the intestinal microbiota in distinguishing patients with sepsis from those without sepsis, based on the difference in the lines of nasal specimens collected.

## INTRODUCTION

Sepsis is a severe illness with a high mortality rate between 29.9% and 57.5%.^1^–3 Despite the establishment of the third international consensus definition of sepsis and septic shock (Sepsis-3) in 2016 (4), there are still many aspects of sepsis that warrant further exploration in order to improve its diagnosis. The evolution of the diagnostic criteria from Sepsis-1 to Sepsis-3 is evidence of this need for continued investigation. Additionally, sepsis diagnostic criteria have shifted from focusing solely on the inflammatory response to also including organ failure caused by infection (4). While considerable progress has been made in the diagnosis of sepsis, no biological indicators with strong sensitivity and specificity have been identified (5). Furthermore, the low culture positivity rate and the presence of few culturable microorganisms limit the diagnosis of clinical sepsis (6). Therefore, the identification of a new, effective, and reliable biomarker for sepsis has long been a goal of researchers.

Previous studies have demonstrated an association between sepsis and dysbiosis of the gut microbiome (7, 8). Literature reports suggest that an imbalance in the intestinal microbiota is a likely cause of sepsis (9). Recent research has indicated that an imbalance in the intestinal microbiota can adversely impact the human immune system by damaging the integrity of intestinal epithelial tissues, thereby creating favorable conditions for the invasion of microbes that cause sepsis (10). Due to the complexity and diversity of intestinal microbiota, humans lack a sufficient understanding of their structural diversity and functional importance (11). It is only in recent years that the development of second-generation sequencing and other technologies has facilitated a deeper understanding of the microbiome (8, 12). Some studies have reported differences in the abundance of certain bacterial genera between patients with sepsis and those without, with a significant increase in pathogenic species such as *Enterococcus* observed in deceased septic patients (13). These findings suggest that these species may serve as potential biomarkers for monitoring during intensive care unit (ICU) hospitalization (13) . However, due to the high complexity of intestinal flora, it is still challenging to describe the disorders and specific characteristics of the intestinal flora (14).

In intensive care unit patients, sepsis is most commonly induced by pulmonary infection (13, 15). Previous studies have reported an association between sepsis and dysbiosis of the lung microbiome (16–18). Recent studies have indicated that the nasal microbial community can reflect the status of deep pulmonary infection due to the similarity in microbial community compositions between the upper and lower airways (19–21). Additionally, acquiring nasal microorganisms is far less invasive than bronchoscopy. Compared to the complexity and diversity of gut microbes, nasal microbes are relatively less complex. This lower complexity provides a foundation for the occurrence of diseases, making it possible to identify common pathogen combinations to build models.

We conducted an observational study by enrolling a cohort of 89 patients diagnosed with sepsis and 65 patients without sepsis. The objectives of this study were to describe the characteristics of the gut and nasal microbiota in both septic and non-septic patients and identify potential microbial biomarkers for diagnosis. Our overarching aim was to characterize the composition of the intestinal and nasal microbiota in septic patients and identify potential microbial biomarkers for the identification of sepsis.

## MATERIALS AND METHODS

### Study design and clinical information collection

The Medical Ethics Committee of the Ethics Committee of Southern Medical University (SMU) approved this study (approval number 2015-GRGLK-002), which involved the collection of clinical information from subjects who provided informed consent . The data collection period extended from January 2015 to May 2017, we desensitized subjects, removed their names and other information, renumbered them, and included them in the study. The information collected included the subject’s sex, age, underlying diseases, catheter placement, antibiotic use, hormone use, inflammatory indicators, and daily blood gas analysis results.

To minimize the impact of antibiotic-related factors, we enrolled non-septic patients who received antibiotic treatment comparable to that administered to the septic patients as the control group.

#### Septic patients

The inclusion criteria were as follows: infection +Sequential Organ Failure Assessment (SOFA score) ≥2 points. The exclusion criteria were as follows: 1) history of significant inflammatory disease other than sepsis; 2) history of lung surgery and tuberculosis; 3) blood transfusion within 4 weeks of enrollment; 4) diagnosis of autoimmune diseases; and 5) enrollment in a blinded drug trial.

#### Non-septic patients

The inclusion criteria were as follows: infection +use of third-generation cephalosporins, quinolones, carbapenems, and/or penicillin plus enzyme inhibitors. The exclusion criteria were as follows: single medication with first- or second-generation cephalosporins and/or macrolides.

Following these inclusion and exclusion criteria, we ultimately enrolled 89 septic patients in the ICU and 65 non-septic patients in the Department of Respiratory and Critical Care Medicine. All subjects provided written informed consent in accordance with the principles of the Declaration of Helsinki.

### Sample collection

For the nasal swab collection, subjects were either seated or placed in a recumbent position to expose the nasal cavity fully. Experienced physicians collected nasal swabs in the morning using disposable swabs. Each swab was inserted into each nostril and rotated ten times.

For the anal swab collection, experienced physicians obtained samples in the morning using disposable swabs. The anal swab was inserted 1–2 cm into the patient’s anus and rotated three times.

All collected samples were temporarily stored in a biological sample transport box and then transferred to a −80°C freezer within 4 hours, where they were stored until the total bacterial DNA was extracted.

### DNA extraction, 16S RRNA gene amplification, and sequencing

Total bacterial DNA magnetic bead extraction kits from Shenzhen Bioeasy Biotechnology Co., Ltd. were used to extract bacterial DNA. PCR amplification targeting the V4-16S rRNA region was performed to prepare 16S rRNA amplicon sequencing samples (22), and the resulting PCR products were mixed at specific ratios using a Qubit fluorometer (InvitrogenTM). Further sequencing was performed using the Illumina HiSeq PE250 platform.

### Bioinformatics analysis and statistical processing

We processed the raw Illumina sequences primarily based on the Greengenes database (23) in QIIME (1.9.1) software (24) , following the same protocol as described in our previous reports (25).

We employed a random forest classification model used for classification, clustering , and regression analyses (26). To construct the model, we utilized the same grouping scheme employed for 16S rRNA gene sequencing. Community data were classified according to the clinical information of the septic patients (metadata). We randomly selected some of the data as the training set and the remaining data as the validation set. The training set was used to train a classifier, and the validation set was used to test the model obtained from the training set (Model) using the R caret package (27). The model aimed to predict outcomes such as sepsis (septic shock and death). During modeling, we randomly divided all samples into ten subsamples for tenfold cross-validations and optimization to obtain the optimal area under the receiver operating characteristic curve (AUC) (28).

Data visualization and statistical analyses were performed using R (3.2.2) statistical software r. The Wilcoxon rank-sum test was used to determine the significance of differences between two groups. Spearman’s rank correlation test was used to analyze the correlations between two variables, while the χ test was employed to compare the ratios of two groups. *P* < 0.05 was considered statistically significant.

## RESULTS

### Patient characteristics

We recruited 157 subjects (89 with sepsis) of both sexes at the Affiliated Hospital of Southern Medical University. The sepsis group consisted of 22 females and 67 males, with participants aged between 22 and 88 years, among which 74 of 89 septic patients had lung infections. The non-sepsis group consisted of 22 females and 46 males, with participants aged between 24 and 94 years, among which 56 of 65 non-septic patients had lung infections, two had tuberculous pleurisy, one had bronchitis, and one had bronchiectasis. No significant differences were found in terms of gender, age, and the number of antibiotic types and days used between the two groups. The patient characteristics are shown in Table 1.

**TABLE 1:** Baseline characteristics of the septic and non-septic patients

Clinical variable	Septic patients	Non-septic patients	Statistical value	*P* value
	(*n* = 89)	(*n* = 68)		
Age (mean ± SD; years)	57.980 ± 16.527	59.4 ± 17.286	t;=0.523	0.602
Sex (male/female; n)	67/22	46/22	χ^2^=1.114	0.291
ICU time (median; interquartile; days)	10 (5.0–24.0)	0 (0.0–0.00)	U = 5386	0.000
Time (median; interquartile; days)	26 (12.0–42.5)	13 (9.00–22.00）	U = 4123.5	1.00E-04
ICU time before sampling (median; interquartile; days)	4.5 (1.0–9.0)	0 (0.0–0.00)	U = 5125	3.09E-14
Time before sampling (median; interquartile; days)	6 (2.0–14.0)	7.00 (4.00–11.00）	U = 2685	0.226
SOFA score (median; interquartile; n)	8 (4.0–10.0)	0 (0–1.0)	U = 6052	0.000
Combination_antibiotic (median; interquartile; n)	2 (1.00–2.00)	2 (1.00–2.00)	U = 2592.5	0.102
Antibiotic_time_before_sampling (median; interquartile; days)	5 (2.00–10)	6 (3.00–10.00)	U = 2571.5	0.106
Hormone (yes/no)	49/40	30/38	χ2 = 1.845	0.174
Nasogastric tube (yes/no)	66/23	10/58	χ2 = 54.554	1.51E-13
Smoking (yes/no)	17/72	25/43	χ2 = 6.138	0.013
Diarrhea (yes/no)	4/85	1/67	Fisher = 0.278	0.390
Diabetes (yes/no)	23/66	8/60	χ2 = 4.821	0.028
Hypertension (yes/no)	39/50	17/51	χ2 = 5.950	0.015

### Nasal microbiota alterations in septic patients compared with non-septic patients

**Fig 1:**
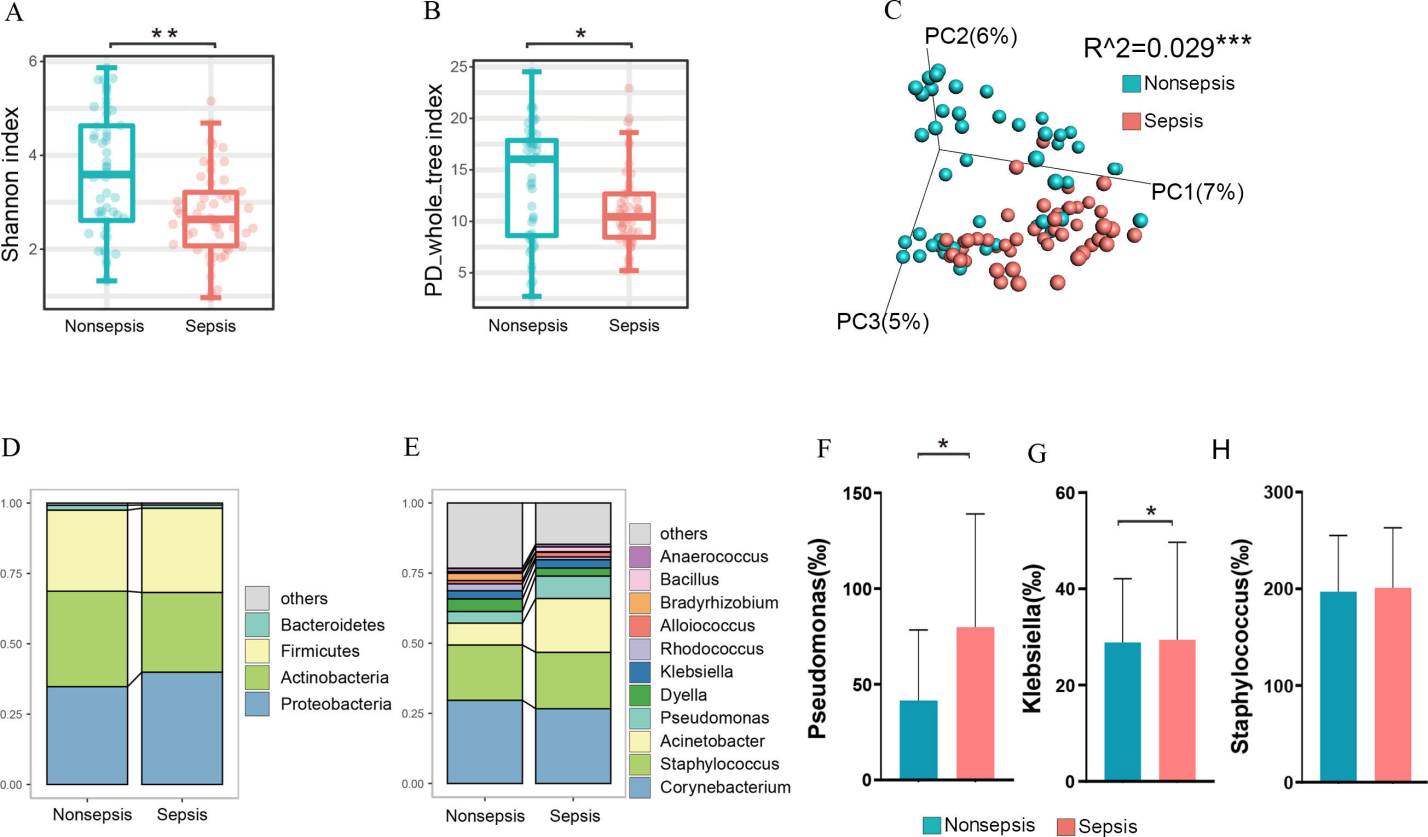
Nasal microbiota alterations in septic patients compared with non-septic patients. (A): The Shannon index of nasal microbiota in septic patients and non-septic patients. (B): The PD whole tree index of nasal microbiota in septic patients and non-septic patients. (C): The nasal bacterial community of septic patients compared with non-septic patients in terms of beta diversity. (D): The key bacteria at the phylum level. (E): The most abundant taxa at the genus level. (F): The abundances of *Pseudomonas* in septic patients compared with non-septic patients. (G): The abundances of *Klebsiella* in septic patients compared with non-septic patients. (H): The abundances of *Staphylococcus* in septic patients compared with non-septic patients.

We utilized 16S rRNA sequencing to examine the microbiota of the gut and respiratory tracts. In comparison to the non-septic patients, the nasal microbiota of the septic patients demonstrated lower community richness according to the Shannon index (*P* = 0.002, Fig. 1A) and PD whole tree index (*P* = 0.019, Fig. 1B). The nasal bacterial community in the non-septic patients differed significantly from that in the septic patients in terms of beta diversity, as indicted by the binary jaccard distance (*P* = 0.001, Fig. 1C).

Given the disparities in nasal microbiota community diversity, we sought to determine which key bacteria were dominant in both patient groups. The dominant phyla were Proteobacteria, Actinobacteria, and Firmicutes (Fig. 1D). In the nasal microbiota of the septic patients, the most abundant taxa were *Corynebacterium*, *Staphylococcus*, *Acinetobacter*, and *Pseudomonas* (Fig. 1E). In comparison to the non-septic patients, the septic patients demonstrated an increase in the abundance of *Pseudomonas* and *Klebsiella* (Fig. 1F and G), a slight decrease in the abundance of *Corynebacterium,* and indiscriminateness in the abundance of *Staphylococcus* (Fig. 1H).

### Gut microbiota in the septic and non-septic patients

**Fig 2:**
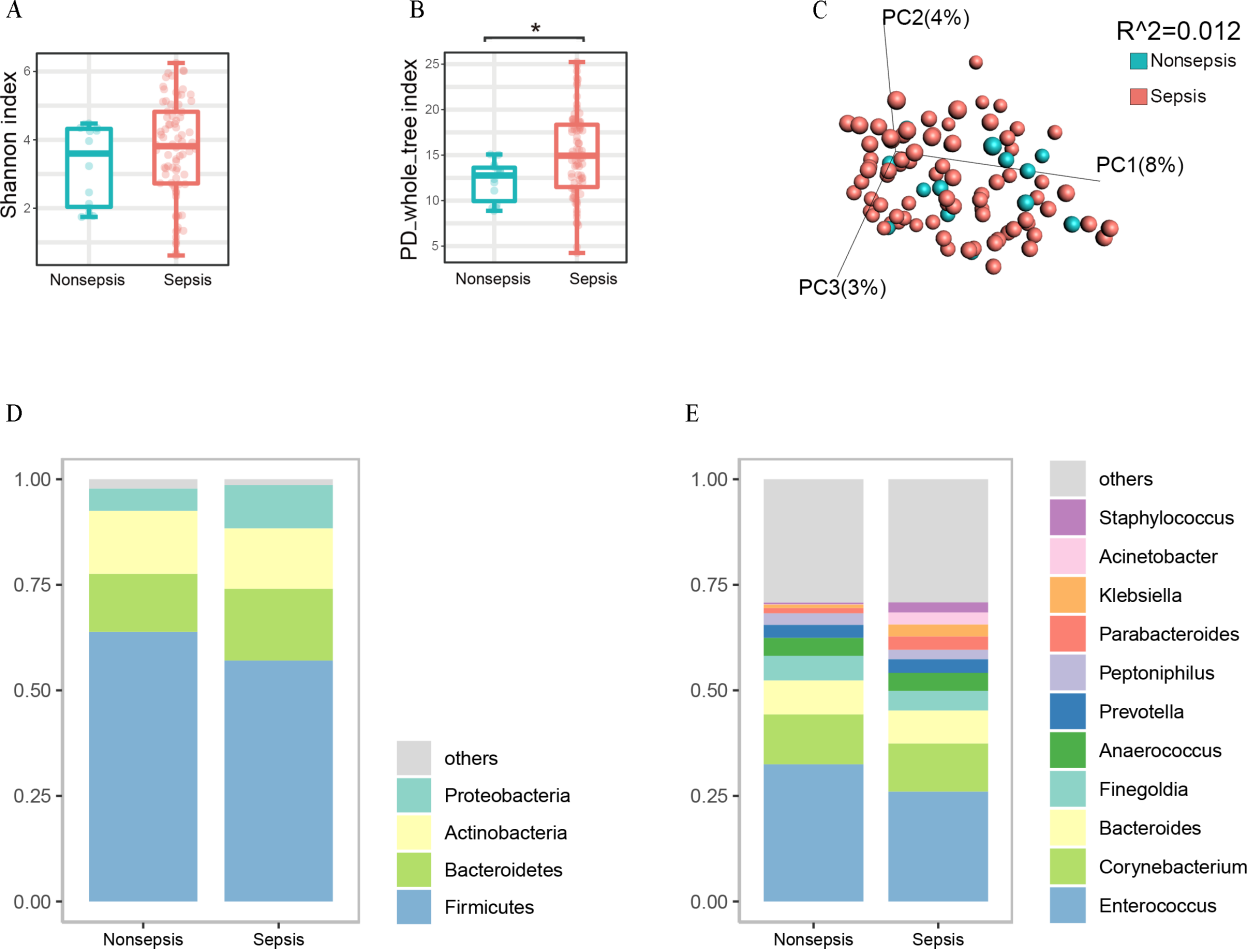
Gut microbiota alterations in septic patients compared with non-septic patients. (A): The Shannon index of gut microbiota in septic patients and non-septic patients. (B): The PD whole tree index of gut microbiota in septic patients and non-septic patients. (C): The gut bacterial community of septic patients compared with non-septic patients in terms of beta diversity. (D): The key bacteria at the phylum level. (E): The most abundant taxa at the genus level. (E): The most abundant taxa at the genus level.

We conducted an analysis of the gut microbiota in the study subjects. Based on the PD whole tree index, we found that the gut microbiota diversity was significantly higher in the septic patients than in non-septic patients (*P* = 0.015, Fig. 2B). Conversely, no significant differences were observed in the Shannon index between the two groups (Fig. 2A). We additionally performed a principal coordinate analysis (PcoA) using the binary jaccard distance, a dimensionality reduction method that illustrates the relationships between samples based on a distance matrix. The result of the PcoA showed no significant differences in gut microbiota between the non-septic and septic patient groups (*P* = 0.126, Fig. 2C).

Furthermore, we evaluated the relative abundances of intestinal bacteria at the phylum and genus level. We found that the most abundant gut bacteria detected in this study belonged to four phyla: Firmicutes, Bacteroidetes, Actinobacteria, and Proteobacteria (Fig. 2D). At the genus level, *Enterococcus* was the most abundant bacteria, followed by *Corynebacterium* and *Bacteroides* (Fig. 2E).

### Machine learning model based on nasal microbiota as the basis for the differences in septic and non-septic patients

**Fig 3:**
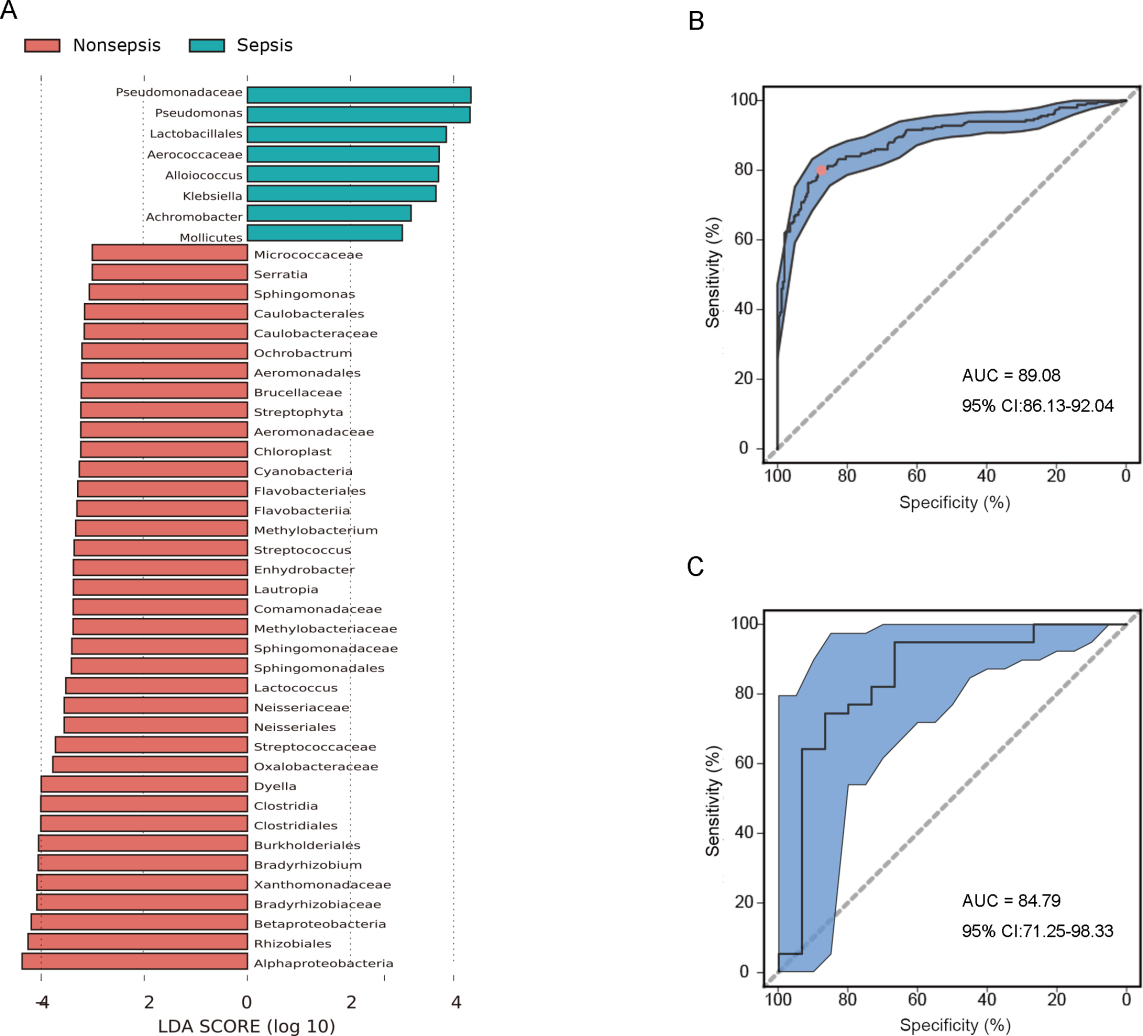
A machine learning model based on nasal microbiota as the basis for the diagnosis of sepsis. (A): Linear discriminant analysis Effect Size (LEfSe) based on the nasal microbiota data between the septic and non-septic patients. (B): A machine learning model using the random forest method based on the nasal microbiota data with an AUC of 89.08 (95% CI: 86.13−92.04). (C): An AUC of 84.79 (95% CI: 71.25−98.33) which was yielded using the nasal microbiota classifier for the diagnosis of sepsis.

The results obtained in this study demonstrated significant differences in the nasal microbiota between the septic and non-septic patients. These differences may provide the basis for the development of a diagnostic tool for sepsis. To this end, a machine learning model using the random forest method was constructed to establish a microecological diagnosis model of sepsis based on the nasal microbiota data. We analyzed 100 nasal microbiota samples from septic and non-septic patients and found that the AUC was 89.08 (95% CI: 86.13–92.04, (Fig. 3B). This result suggested that the nasal microbiota could be used to distinguish between septic and non-septic patients. Additionally, we employed Linear discriminant analysis Effect Size (LEfSe) to identify the bacterial taxa that differed significantly between septic and non-septic patients. The result revealed increased abundances of Pseudomonadaceae and *Pseudomonas* , as well as reduced abundances of *Alphaproteobacteria*, *Rhizobiales*, *Prevotella*, *Betaproteobacteria*, *Bradyrhizobiaceae*, *Xanthomonadaceae*, *Bradyrhizobium*, *Burkholderiales*, *Clostridiales*, *Clostridia*, and *Dyella* (Fig. 3A).

### Model validation indicated that the nasal microecological diagnosis prediction model was effective

Given the robust predictive performance of the random forest nasal microbiota classifier, we conducted further analysis by classifying an additional 39 nasal bacterial species from septic patients and 15 nasal bacterial species from non-septic patients. This was done to better evaluate the potential of using the nasal microbiota classifier as a diagnostic tool for sepsis. The result of this analysis showed that the AUC was 84.79 (95% CI: 71.25–98.33; Fig. 3C), indicating that the nasal microecological diagnosis prediction model was effective for diagnosing sepsis.

### The gut microecological diagnosis prediction model had poor predictive performance

**Fig 4:**
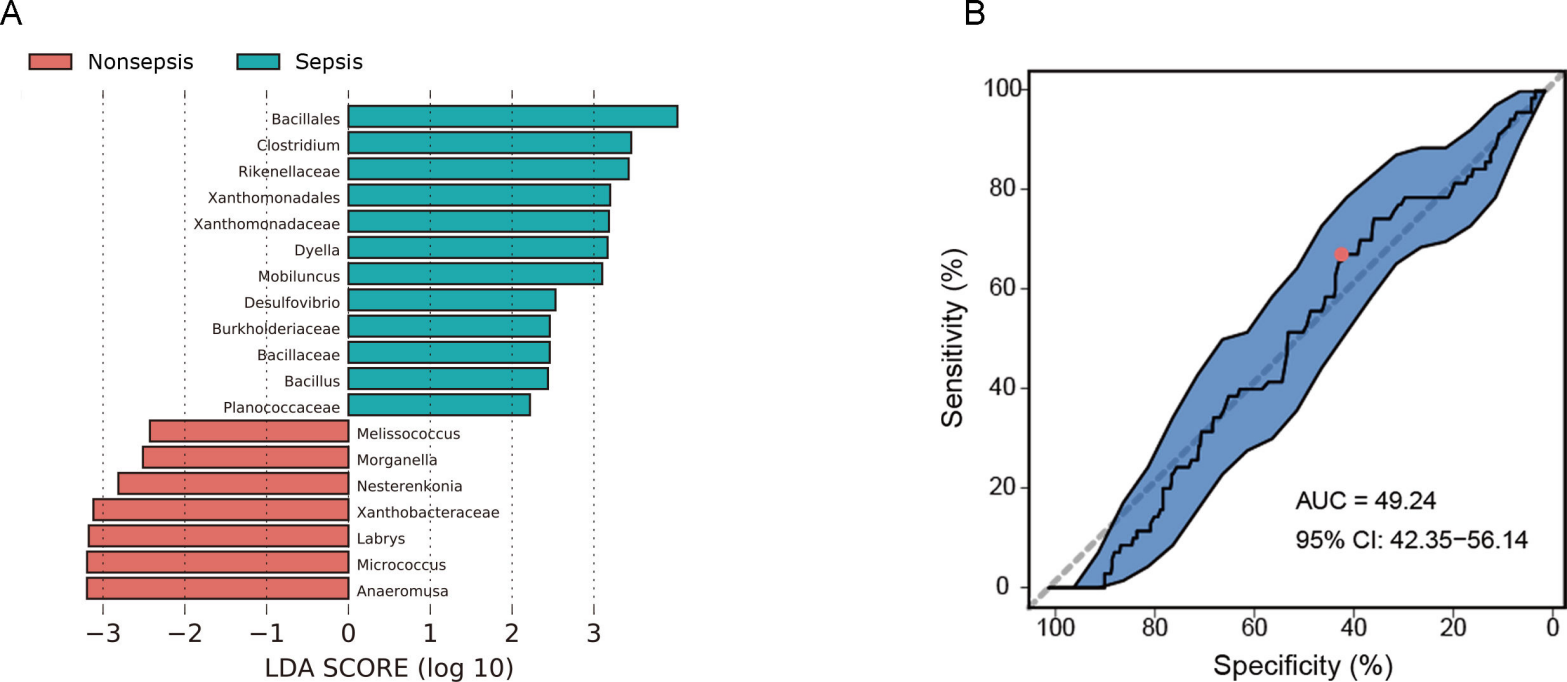
The gut microecological prediction model of sepsis. (A): LEfSe based on gut microbiota between the septic patients and non-septic patients. (B): An AUC of 49.24 (95% CI: 42.35–56.14) was achieved by a random forest classifier using the intestinal microbiota data.

LEfSe analysis revealed only minor differences between septic and non-septic patients, with increased abundances of *Bacillales*, *Clostridium*, and *Rikenellaceae* and reduced abundances of *Anaeromusa* and *Micrococcus* (Fig. 4A). In an attempt to distinguish between septic and non-septic patients using the intestinal microbiota data, we constructed a random forest classifier using 80 septic and 14 non-septic patient specimens. However, the model only achieved an AUC of 49.24 (95% CI: 42.35–56.14, Fig. 4B), indicating weak predictive performance in distinguishing septic and non-septic patients based on the intestinal microbiota.

## DISCUSSION

At present, the diagnosis of sepsis remains challenging due to the lack of specificity in the diagnostic methods used. Sepsis is a broad term applied to an incompletely understood process, and there are currently no simple and unambiguous clinical criteria or biological, imaging, or laboratory features that uniquely identify sepsis (4). The lungs are the most common source of sepsis (29). While numerous studies have shown that the nasal microbial community can reflect the deep pulmonary infection status, it remains unclear whether nasal microbiota can be used to diagnose sepsis . To address this issue, we conducted 16S rRNA sequencing and utilized a machine learning approach. Our results showed that sepsis was associated with a distinct nasal microbiota signature that could distinguish septic patients from non-septic patients. Specifically, the most abundant genera in the nasal microbiota of the septic patients were *Corynebacterium*, *Staphylococcus*, *Acinetobacter*, and *Pseudomonas*. Compared with those in the non-septic patients, *Pseudomonas* was one of the most significantly abundant genera , which is usually related to pneumonia (30, 31). *Klebsiella pneumoniae* causes healthcare-associated pneumonia worldwide (32, 33)*.* Polymicrobial, *Streptococcus pneumoniae*, and *Staphylococcus aureus* were the most common bacteria in lower respiratory infections (34). Thus, our study provides a novel approach based on the nasal microbiota to identifying sepsis in the ICU environment.

We observed little differences in intestinal microbiota between septic and non-septic patients, despite the gut being another important source of sepsis. The similarity in the gut microbiota of septic and non-septic patients may be attributed to the administration of antibiotics during hospitalization, which has a significant impact on intestinal microbiota (13, 35–40). To address this issue, we collected control samples from non-septic patients who were administered the same antibiotics used in the septic patients. Our findings showed that intestinal microbiota in both septic and non-septic patients changed dramatically with the administration of a large number of antibiotics. In the septic patients, the main enriched phyla were Actinomycetes, Proteobacteria, and Firmicutes, while in the non-septic patients, they were Firmicutes, Bacteroides, and Actinomycetes (41). Interestingly, we found that the changes in the nasal microbiota of septic patients were smaller than those in the gut microbiota, suggesting that a large number of antibiotics have less influence on the nasal microbiota than on the intestinal microbiota. Given the widespread use of antibiotics in the ICU, the nasal microbiota may be more suitable than intestinal microbiota for identifying septic patients.

Compared to the complex and diverse range of gut microbes, nasal microbes are relatively less complex. Low α-diversity indicates a low number of microbial species in a single sample. The gut is known to harbor hundreds or thousands of microbes, whereas the nasal flora is about half of that. Low β-diversity refers to little variation between individuals, i.e., the nasal flora among different individuals contains more common species than the intestinal flora. This relatively low complexity provides a common foundation for disease occurrence, making it possible to identify common pathogen combinations for building models. This relatively low complexity provides a common foundation for disease occurrence, making it possible to identify common pathogen combinations for building models. Furthermore, the relatively simple composition of the nasal microbiota suggests that it will be easier to establish rapid PCR diagnostic technology based on this model in the future. On the other hand, due to the relatively low complexity, traditional culture-based and PCR-based studies provide more effective information, which provides clues for our understanding and interpretation of the model.

In summary, the important clinical significance of this study is that it compared the intestinal and nasal microbiota of patients with sepsis and those without sepsis and determined that the nasal microbiota is more effective than the intestinal microbiota in distinguishing patients with sepsis from those without sepsis, based on the difference in the lines of nasal specimens collected. A machine learning model based on nasal microbiota provides a basis for identification of sepsis. The model verification showed that the prediction model of nasal microecological diagnosis was effective in providing a reference for the clinical application of nasal microflora in the identification of sepsis in ICU patients. There are some limitations to the study: in view of the fact that extensive use of broad-spectrum antibiotics can significantly alter the intestinal flora of severely ill patients (42, 43). The diversity of intestinal microbiota in patients with critical sepsis decreased significantly, and the bacterial composition was dominated by multidrug-resistant bacteria (44). We acknowledge the complexities associated with microbiological analysis in the context of prior antibiotic use, which indeed complicates the microbial landscape. However, the reality of clinical practice often involves patients receiving antibiotics before a definitive diagnosis of sepsis is made, particularly those who are critically ill and subsequently admitted to the ICU. It is pertinent to note that our study aimed to reflect the real-world clinical scenario where most sepsis patients have been pretreated with antibiotics by the time of ICU admission. This pretreatment, while challenging, provides a unique insight into the microbiota dynamics post-antibiotic intervention, which is a critical aspect of sepsis management and its prognostic evaluation. The complexity introduced by antibiotics is not merely a limitation but an integral part of the sepsis pathology that deserves attention for its implications on patient outcomes and disease prognosis (45). Looking forward, we suggest the potential for further research, possibly through animal models or larger patient cohorts, to deepen our understanding of the role of microbiota in sepsis beyond the antibiotic effect.
